# Supplementary figures and images for: Primer extension coupled with fragment analysis for rapid and quantitative evaluation of 5.8S rRNA isoforms
Source: PLoS One. 2021 Dec 21;16(12):e0261476. doi: 10.1371/journal.pone.0261476 (PMC8691633; doi:10.1371/journal.pone.0261476)

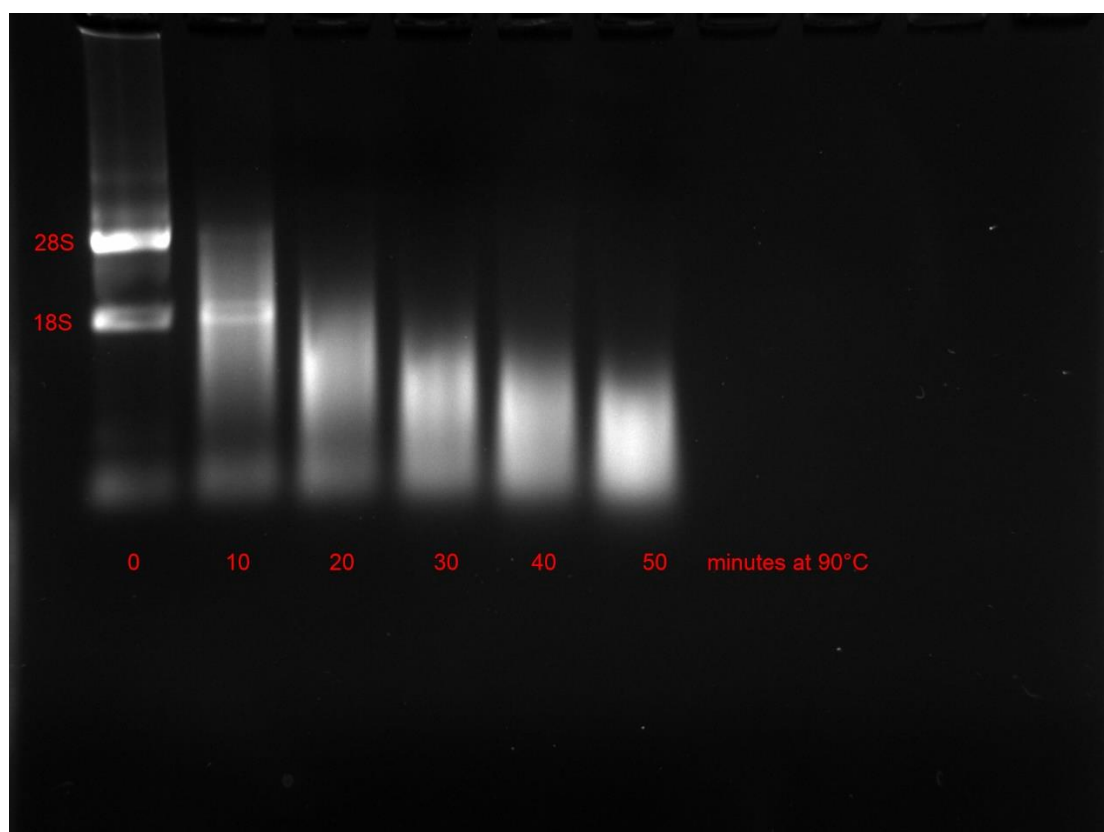

Fig. 4B, image obtained by UV Trans Illumination (ChemiDoc), exposure time 0.247 seconds.

Supplement: S1 Raw images — (PDF) [file pone.0261476.s001.pdf]
