# Supplementary material for: Primer extension coupled with fragment analysis for rapid and quantitative evaluation of 5.8S rRNA isoforms
Source: PLoS One. 2021 Dec 21;16(12):e0261476. doi: 10.1371/journal.pone.0261476 (PMC8691633; doi:10.1371/journal.pone.0261476)
Supplement: Attachment — Submitted filename: Response to Reviewers2.docx [file pone.0261476.s003.docx]

Academic Editor’s comments:

Legend for Fig.1A should define the short names for the three isoforms.

We amended the figure legend

Line 28 and 76, ”two 5.8S isoforms” should be “three 5.8s isoforms”.

We corrected the errors
